# Normative Data for 84 UK English Rebus Puzzles

**DOI:** 10.3389/fpsyg.2018.02513

**Published:** 2018-12-13

**Authors:** Emma Threadgold, John E. Marsh, Linden J. Ball

**Affiliations:** ^1^School of Psychology, University of Central Lancashire, Preston, United Kingdom; ^2^Department of Building, Energy and Environmental Engineering, University of Gävle, Gävle, Sweden

**Keywords:** problem solving, insight, rebus, norming, test validation

## Abstract

Recent investigations have established the value of using rebus puzzles in studying the insight and analytic processes that underpin problem solving. The current study sought to validate a pool of 84 rebus puzzles in terms of their solution rates, solution times, error rates, solution confidence, self-reported solution strategies, and solution phrase familiarity. All of the puzzles relate to commonplace English sayings and phrases in the United Kingdom. Eighty-four rebus puzzles were selected from a larger stimulus set of 168 such puzzles and were categorized into six types in relation to the similarity of their structures. The 84 selected problems were thence divided into two sets of 42 items (Set A and Set B), with rebus structure evenly balanced between each set. Participants (N = 170; 85 for Set A and 85 for Set B) were given 30 s to solve each item, subsequently indicating their confidence in their solution and self-reporting the process used to solve the problem (analysis or insight), followed by the provision of ratings of the familiarity of the solution phrases. The resulting normative data yield solution rates, error rates, solution times, confidence ratings, self-reported strategies and familiarity ratings for 84 rebus puzzles, providing valuable information for the selection and matching of problems in future research.

## Introduction

Problem solving involves thinking activity that is directed toward the achievement of goals that are not immediately attainable (e.g., Newell and Simon, 1972). It is a central aspect of human cognition that arises across a range of contexts, from everyday activities to the attainment of major scientific advancements and the achievement of important technological, cultural, and artistic developments. Although problem solving can be fairly mundane (e.g., deciding what to make for your evening meal) it can also lead to solutions that are highly creative (e.g., a delicious new dish prepared by a master chef). This latter kind of “creative problem solving” is distinguished from other types of problem solving in that it involves the generation of solutions that are both original *and* effective, with the sole presence of either attribute being insufficient for a solution to be deemed creative (see Runco, 2018). Not surprisingly, creative problem solving is held in especially high regard in all areas of real-world practice.

Research on creative problem solving has burgeoned over the past 20 years, with a traditional assumption being that people solve such problems in one of two different ways, that is, either (i) through *analytic* processes, which involve conscious, explicit thinking that takes the solver closer to a solution in a slow, step-by-step manner (e.g., Fleck and Weisberg, 2004; Ball and Stevens, 2009); or (ii) through *insight* processes, which involve non-conscious, implicit thinking that gives rise to a sudden and clear realization of how to make progress toward a solution (e.g., Sternberg and Davidson, 1995; Bowden and Jung-Beeman, 1998, 2003a; Jung-Beeman et al., 2004). According to the latter view, such flashes of insight are typically characterized as involving a major change in the representation of a problem, arising from largely tacit processes of problem elaboration, recoding or constraint relaxation (e.g., Ohlsson, 1992, 2011; Knoblich et al., 1999; see also Bowden et al., 2005).

Notwithstanding the possibility that creative problem solving can, in principle, occur in two distinct ways (i.e., either via explicit, analytic processes or via implicit, insight processes) the emerging consensus is that a good deal of the time people probably deploy a mix of both conscious analysis and non-conscious insight when tackling creative problems (e.g., Barr et al., 2014; Sowden et al., 2014; Gilhooly et al., 2015; Weisberg, 2015, 2018; Barr, 2018). This move away from polarized views of creative problem solving as involving *either* analytic processes *or* insight processes marks an important change in recent theorizing, which over the past couple of decades has tended to become sidetracked by rather narrow and somewhat esoteric debates focused on a very limited set of tasks and paradigms.

The welcome emergence of more nuanced and encompassing theories of creative problem solving has arguably been fueled not only through improved theory-driven experimentation (including neuroscientific studies; for a recent review see Shen et al., 2017), but also through the availability of a greater variety of problem-solving tasks that can be used by researchers in laboratory-based studies of problem-solving phenomena. This means that nowadays researchers are not just reliant on so-called “classic” insight tasks that often have their origins in Gestalt studies of problem solving (e.g., Duncker, 1945, candle problem or Maier, 1930, nine-dot problem), but that they can also make use of many other problems that may be solved to varying degrees by analysis or insight, such as remote associate tasks (RATs) (e.g., Mednick, 1968), matchstick algebra problems (e.g., Knoblich et al., 1999), magic tricks (e.g., Danek et al., 2014a,b) and rebus puzzles (e.g., MacGregor and Cunningham, 2008; Salvi et al., 2015), which are the focus of the present paper.

Classic insight problems suffer from a number methodological issues that have arguably limited their value in advancing an understanding of creative problem solving (for relevant arguments see Bowden and Jung-Beeman, 2003a; MacGregor and Cunningham, 2008). Most notably, there is a restricted pool of such classic insight problems from which researchers can draw, which means that studies using these problems often involve only a small number of items. In addition, classic insight problems can be very difficult to solve, with very few participants achieving a correct solution without some sort of hint being provided. Moreover, problem-solving times can be lengthy, often taking up to 10 min per problem. Classic insight problems are also heterogeneous and prone to being influenced by confounding variables (e.g., the amount of time that is available for solution generation itself is an important confounding factor that is often overlooked in theorizing; but see Ball et al., 2015). These problems may also yield ambiguous solutions that are difficult to quantify.

As an alternative to classic insight problems, researchers have turned in recent years toward the extensive use of compound remote associates (CRA) problems, which are conceptual descendants of the RAT first developed by Mednick (1968). CRA problems involve presenting participants with three words (e.g., pine, crab, sauce) for which they are required to produce a solution word which, when combined with the three words, generates three compound words or phrases (i.e., pineapple, crab apple, apple sauce). CRA problems have significant advantages over classic insight problems. Although variation of problem difficulty exists within CRA sets (Bowden and Jung-Beeman, 2003a; Salvi et al., 2015) they are comparatively easy to solve, fast to administer, more resistant to potentially confounding variables and typically yield unambiguous solutions (Bowden and Jung-Beeman, 2003a; MacGregor and Cunningham, 2008; but see Howe and Garner, 2018). Importantly, too, it is possible to construct a large number of CRA problems, as has recently been demonstrated by Olteteanu et al. (2017), who used computational methods to generate a repository of around 17 million American English CRA items based on nouns alone and meeting tight controls. Furthermore, CRA problems can be presented in compressed visual areas, rendering the problems suitable for electroencephalography (EEG; e.g., Bowden and Jung-Beeman, 2003a,b; Sandkühler and Bhattacharya, 2008) and functional magnetic resonance (fMRI; e.g., Bowden and Jung-Beeman, 2003a,b) procedures. In addition, CRA problems allow for control over stimulus presentation and response timing (e.g., Bowden and Jung-Beeman, 2003a) and lend themselves well to priming paradigms in which primes (e.g., Howe et al., 2016) solution hints (e.g., Smith et al., 2012) or solution recognitions can be presented across or within hemispheres (e.g., Bowden and Jung-Beeman, 2003b).

In the present paper we focus on rebus puzzles (e.g., MacGregor and Cunningham, 2008; Salvi et al., 2015), which are starting to feature more commonly in problem-solving research and have many of the benefits of CRAs, as well as some additional advantages. Rebus puzzles involve a combination of visual, spatial, verbal, or numerical cues from which one must identify a common phrase or saying. As an example, the rebus problem “BUSINES,” when correctly interpreted, yields the common phrase “Unfinished Business.” Such rebus problems have been used in research on creative problem solving-processes such as studies of fixation and incubation phenomena (Smith and Blankenship, 1989), with rebus problem-solving success also having been shown to be positively correlated with performance on remote associate problems, whilst being independent of general verbal ability (MacGregor and Cunningham, 2008).

Rebus puzzles are relatively easy to present to participants and have only single “correct” answers, which means that responses are straightforward to score. Importantly, however, the problems are moderately challenging to solve, although they are often solvable with persistent effort. The difficulty of rebus puzzles may arise, in part, from there being many ways in which they can be tackled (cf. Salvi et al., 2015), but may also be a consequence of the problem information initially *misdirecting* solution efforts because the solver draws upon implicit assumptions derived from the experience of normal reading (Friedlander and Fine, 2018, similarly suggest that normal reading may engender misdirection when solving cryptic crossword clues). Such self-imposed constraints may lead solvers to reach a point of *impasse*, where solution progress is not forthcoming, with such impasse needing to be circumvented by problem restructuring (see MacGregor and Cunningham, 2008; Cunningham et al., 2009). The challenges for solving rebus puzzles that arise from tacit, self-imposed assumptions can readily be seen in the rebus example “CITY,” whose solution is “capital city.” The font of the presented text is a superficial feature that would usually be ignored in normal reading, despite potentially carrying figurative meaning in the context of a rebus puzzle. Indeed, the difficulty of a rebus problem is believed to be a function of the number of implicit assumptions that need to be broken (MacGregor and Cunningham, 2008, 2009).

Another factor that makes rebus problems useful in problem-solving research is the observation that solvers often cannot report the details of the preceding processing that led to a solution, which is especially likely when such solutions are accompanied by an “Aha!” experience that is suggestive of an insight-based problem-solving process (MacGregor and Cunningham, 2008). Notwithstanding the fact that rebus puzzles can be solved via implicit, insight processes, there is also evidence that they are open to solution via analysis as well or a varying combination of both analysis and insight (MacGregor and Cunningham, 2008, 2009).

In sum, rebus puzzles offer a means by which a large pool of homogenous problems of different difficulty can be administered within a single session in order to investigate the processes of analysis and insight that underpin creative problem solving. Such rebus puzzles are rapid to administer and relatively under-represented in the problem-solving literature in comparison to tasks such as CRA problems. Despite the increasing use of rebus puzzles in problem-solving research, there exists very limited normative data relating to such problems in relation to their solution rates, solution times and phenomenological characteristics, with current norming studies being restricted (as far as we are aware) to the validation of a set of Italian rebus puzzles (Salvi et al., 2015). The lack of normative data is problematic given that rebus puzzles are linguistically context dependent, relating, as they do, to common words, sayings or phrases that exist in a particular language, including idiomatic expressions that have become culturally conventionalized. Language-specific normative data are, therefore, vital for advancing the use of rebus puzzles in problem-solving studies so that researchers can have confidence that the problems that they select for their experiments have desired characteristics to enable specific research questions to be studied.

To address the absence of normative data for English rebus puzzles, this paper presents normative data for 84 rebus items that are underpinned by common United Kingdom (UK) English phrases or sayings. The normative data that we obtained provide details of typical solution rates, error rates, and correct solution times (seconds) as well as standard deviations for all solution times. In addition, we obtained ratings of participants' confidence in their solutions, their familiarity with the solution phrases as well as a self-report measure of the extent to which participants felt that they had solved the problem via a process of analysis vs. insight. The latter data were elicited to align with the emerging theoretical consensus that it is useful to view creative problem solving as involving a mix of processes that fall along a continuum ranging from analysis to insight.

We further note that an inspection of rebus puzzles revealed to us that there are several specific sub-types that involve very similar solution principles. This was also highlighted in the set of Italian rebus puzzles reported by Salvi et al. (2015), in which they identified 20 categories for the subset of rebus problems. On inspection of the UK English rebus puzzles, we categorized the puzzles into substantially fewer categories based on an observation of the specific solution principles that underpin these rebus items. We categorized the 84 rebus puzzles that we wished to norm into six specific categories relating to their structure and the types of cues necessary to solve each problem.

## Method

### Participants

The study involved 170 participants in total (125 female) with an age range of 19 to 70 years (*M* = 36 years-old, *SD* = 12 years). Participants received £3 in exchange for 30 min participation time and were recruited via the survey recruitment website “Prolific Academic.” Participants completed one rebus set each. All participants were UK nationals and native English speakers. This study was carried out in accordance with the recommendations of the British Psychological Society Code of Human Research Ethics. The protocol was approved by the Psychology and Social Work Ethics Committee (Ref: 397) at the University of Central Lancashire, UK. All subjects gave written informed consent in accordance with the Declaration of Helsinki.

### Design

A total of 84 rebus puzzles were collated and divided into two equal sets, with 42 rebus puzzles per set (see Appendix A in Supplementary Material for the Set A items, Appendix B in Supplementary Material for the Set B items and Appendix C in Supplementary Material for three practice items that were used in the study). For each rebus puzzle, a normative solution rate and mean solution time (in seconds) was obtained. The maximum available solution time per item was 30 s. The dependent variables were the solution rate and solution time for each rebus, an error rate, a measure of confidence in the accuracy of the response to each rebus, and a measure of the extent to which each answer was solved via a process of analysis or insight. The confidence measure and the measure of analysis/insight phenomenology were each elicited using continuous sliding scales that participants used to register a response, resulting in scores ranging from 1 to 100. Thus, a higher score indicated a more confident response on the confidence scale and a more “insight-like” response on the analysis/insight scale. Furthermore, each rebus puzzle was allocated to one of six categories based on their underpinning solution principles, with these categories having been developed for initial classification purposes (please refer to the Materials section below for a discussion of the development of these categories).

### Materials

#### Rebus Puzzles

An initial set of 186 rebus puzzles were selected from copyright-free sources on the internet. It was ensured that the rebus puzzles all related to familiar UK English phrases, with the removal of any specifically American phrases. On inspection of the set of rebus items, it became clear that there were many common structural features across the puzzles. Therefore, six puzzle categories were developed to which each rebus item could be allocated so as to ensure that different types of rebus were presented in a balanced manner across item sets (see Appendices A, B in Supplementary Material). The six rebus categories that were developed are as follows: (1) a word, picture or number over another word, picture or number (for an example item see Appendix A in Supplementary Material, Item 1—“*feeling on top of the world”*); (2) a word, picture or number under another word, picture or number (see Appendix A in Supplementary Material, Item 5—“*try to understand”*); (3) a word presented within another word (see Appendix A in Supplementary Material, Item 6—“*foot in the door”*); (4) a play on words with numbers (see Appendix A in Supplementary Material, item 16—“*forty winks”*); (5) imagery (see Appendix A in Supplementary Material, Item 20—“*half hearted”*); and (6) spatial (see Appendix A in Supplementary Material, Item 36—“*parallel bars”*).

Drawing from the initial set of 186 rebus puzzles, each puzzle was allocated by two independent judges to one of the six constructed rebus categories. An inter-rater reliability analysis was then undertaken utilizing the Kappa statistic (Viera and Garrett, 2005) to determine the overall consistency in rebus categorization between the two judges. There was a statistically significant moderate agreement between the two judges, κ = 0.59 (95% CI = 0.50 to 0.67, *p* < 0.001). It was also evident from viewing the rebus puzzles that a number of them might be deemed to cross two or more categories. To account for this, and utilizing the Kappa scores, rebus items were selected for the norming study only when category agreement had been reached between the two judges. This resulted in a reduced pool of 126 rebus puzzles from the initial pool of 186. From this pool of 126 puzzles, 3 were selected to serve as practice items (see Appendix C in Supplementary Material) and 84 puzzles were randomly selected for norming, with 42 being allocated to Set A and 42 to Set B (see Table 1 for details). The number of puzzles that were allocated to each puzzle category within Set A and Set B were balanced where possible. A number of puzzle categories were more commonly represented than others, with items falling into the imagery category being most prevalent, although it should be noted that this category also involves more varied items than the other categories. It can also be seen from Table 1 that Categories 1 and 2 had the lowest representation in Sets A and B, although this relative under-representation may serve to allay concerns that at an abstract level the solution principle underpinning puzzles in these two categories is very similar.

**Table 1:** The number of rebus items per puzzle category for Set A and Set B.

**Puzzle category**	**Rebus set A**	**Rebus set B**	**Total**
1. A word, picture or number, over another word, picture or number	4	3	7
2. A word, picture or number, under another word, picture or number	1	2	3
3. A word presented within another word	7	7	14
4. A play on words with numbers	7	7	14
5. Imagery	15	15	30
6. Spatial	8	8	16
Total	42	42	84

A further three rebus puzzles were selected as practice problems (see Appendix C in Supplementary Material). These problems served as practice items for both Set A and Set B items. These practice puzzles were chosen from the pool of problems for which an agreement had not been reached on a category, and had answers as follows: “all over again,” “once upon a time” and “long johns” (Appendix C in Supplementary Material). Problems for which an agreement had not been reached were selected as practice items so as not to provide a specific strategic advantage to the solving of any category of rebus puzzle in the norming study. Each participant received the same three practice problems in a fixed order, regardless of the rebus set that they had been allocated.

#### Phrase Familiarity Task

In order to solve rebus puzzles, a particular phrase or saying must be identified from the pictorial, number and word representation provided. A rebus phrase familiarity task was developed to test participants' familiarity with the phrases (or answers) of each rebus puzzle. Following completion of the full set of rebus items, participants were presented with the phrases from the 126 Rebus where a category agreement had been reached, and a further 26 “pseudo phrases” developed by the experimenters (see Appendix D in Supplementary Material). The pseudo phrases were based on existing and well-known common UK English phrases. For example, “knock on metal” is a variant of the common phrase “touch wood.” These phrases therefore had an element of plausibility, whilst not being a common phrase or saying in UK English. The aim of the pseudo phrases was to ensure participants' task engagement during the phrase familiarity rating task and thereby counteract any tendency toward purely confirmatory responding. Each phrase was presented to participants, and they were asked to respond with “yes” if the phrase was familiar to them, and “no” if the phrase was not familiar. Participants were informed that familiarity might stem from the experiment or from encountering these phrases in everyday life.

### Procedure

Each participant completed the experiment individually and remotely via a desktop PC, laptop computer, or tablet. Participants read an information sheet and indicated consent to participate in the experiment before proceeding. Each participant completed only one set of rebus puzzles (Set A or Set B). The experiment was constructed using Qualtrics experimental survey software and deployed through Prolific Academic, a survey recruitment platform. Each participant completed the set of rebus puzzles initially, followed by the phrase familiarity task.

The task instructions were presented on the screen for participants to read through prior to commencing the computerized rebus task. Participants were informed that they would be presented with a combination of words, pictures, or numbers on the computer screen and that their task was to identify the common word, phrase or saying represented by these words, pictures or numbers. Participants were also informed that they would complete 42 rebus puzzles in the study in addition to tackling three practice items to begin with. On completion of the three practice puzzles the answers were provided. This practice phase helped to ensure that participants were familiar with the general nature of rebus puzzles as well as with the response requirements of the study. The three practice items were identical for Set A and Set B. For each set of rebus puzzles, the presentation of the items was randomized by the Qualtrics programme.

All rebus puzzles were presented one at a time in black and white and were based within a square at the center of the computer screen covering an area of approximately 10 cm by 10 cm. The instructions required participants to read the rebus puzzle carefully, consider their answer, and when they had generated their final answer to input it in the text box provided. A maximum of 30 s was provided to view each rebus puzzle and generate and input an answer to it. The participant was able to see the timer display with the 30 s time limit. The clock was stopped when the participant moved onto the following page. This was to ensure that further thinking time was not taken when inputting an answer to the problem. If an answer was not provided within this 30 s time limit, the programme automatically advanced onto the next page.

Following each rebus puzzle a screen appeared asking participants to rate their confidence in the accuracy of their answer on a sliding scale ranging from 1 to 100, where 1 was labeled as “not at all confident” and 100 was labeled as “very confident.” Participants moved the cursor to the appropriate point on the scale to reflect their confidence in their answer for that problem. A “not-applicable” box was also provided for each rebus puzzle and participants were asked to select this box to register a response to the confidence question in all cases where they had not given an answer to the preceding puzzle.

Following the confidence judgment question, participants were next asked to provide a rating to indicate their perceived solution strategy, that is, whether they felt they had solved the preceding rebus puzzle more by analysis or more by insight (i.e., “*Did you feel as if the problem was solved more by insight or more by analysis?”*). It was emphasized that insight and analysis are two ends of a continuum, and therefore participants were asked to indicate if their answer was more “analytic-like,” or “insight-like” by responding on a sliding scale. An “insight” response was described as the following: “*Insight means that the answer suddenly (i.e., unexpectedly) came to your mind while you were trying to solve the problem, even though you are unable to articulate how you achieved the solution. This kind of solution is often associated with surprise exclamations such as ‘A-ha!’.”* An analysis response was described as the following: “*Analysis means that you figured out the answer after you deliberately and consciously tested out different ideas before you found the right phrase or saying. In this case for instance, you are able to report the steps that you used to reach the solution.”* The ends of the response scale in relation to the analysis vs. insight question were alternated and counterbalanced across participants. A “not-applicable” box was also provided for participants to select in those cases where they had not given an answer to the preceding rebus puzzle. Participants were forced to respond by either moving the cursor from the mid-way point (50) on the sliding scale, or by selecting the “not applicable” box before proceeding to the next page.

On completion of the 42 rebus puzzles, participants completed a phrase familiarity task. This involved them rating a list of 152 phrases that were presented in a fixed, sequential order. In this task the participants were presented with the phrases from the 126 rebus puzzles for which a category judgment agreement had been reached by the raters, along with a further 26 “pseudo” rebus phrases (see Appendix D in Supplementary Material). Pseudo phrases were utilized to ensure that a number of phrases were likely to elicit a “no” response to the familiarity question. For each phrase, word, or saying, participants were asked to respond “yes” to indicate that the phrase was familiar to them, and a “no” to indicate that the phrase was not familiar. At the end of the experiment participants were debriefed and thanked for their participation time.

## Results

### Fundamental Performance Characteristics of Each Rebus Puzzle

Performance data were collated for the 84 rebus puzzles across the two sets of items. Each participant completed only one set of 42 rebus puzzles, with 85 participants completing the 42 Set A items and another 85 participants completing the 42 Set B items. For each rebus puzzle we calculated the number of correct solutions and the number of incorrect solutions that had been provided by the 85 participants. This allowed us to calculate the percentage of correct solutions for a particular rebus item, which we subsequently refer to as the *solution rate*. Note that a response was counted as being an “incorrect solution” if an answer to the rebus puzzle had been provided by a participant that was not the correct phrase or saying. For example, in response to the rebus puzzle “try to understand,” incorrect solutions included “try to stand up” and “try to stand divided.” A “don't know” response, or no attempt at an answer, was *not* counted as an “incorrect solution,” but was instead designated as being a null response.

In addition, for each correctly solved rebus puzzle we calculated the mean and standard deviation for its solution time (out of a maximum of 30 s). The solution time was the time spent on the rebus puzzle page, including the time to input the answer. This was to ensure that any additional time spent contemplating the answer during the process of typing, was accounted for in the timing analysis. When 30 s had elapsed, the programme progressed to the next rebus puzzle. Furthermore, for each rebus puzzle we calculated a mean confidence rating for correct solution responses, where ratings could range from 1 (not at all confident) to 100 (very confident). For the insight vs. analysis rating, we again determined for each correctly solved rebus item the extent to which it was deemed to have been solved more by insight or more by analysis. The measurement scale ranged from 1 (analysis) to 100 (insight).

The various performance measures calculated for each rebus puzzle are presented in Table 2, with rebus items organized in the table in descending order of solution rate (i.e., from the easiest to the most difficult). As shown in Table 2, it is evident that the 84 rebus puzzles vary greatly in terms of their difficulty, with solution rates ranging from 95.29 to 0%, and with mean solution times for correct responses ranging from 8.68 to 22.64 s. We contend that the variability in both solution rates and solution times for this set of rebus puzzles is of great benefit for the selection of rebus stimuli for use in future experimental research. We note, in particular, that there are 50 rebus puzzles with a solution rate between 20 and 80%, which provides a good number of items for future use even when those puzzles are discounted that might be viewed as demonstrating either floor or ceiling effects. We also note that the performance data in Table 2 provide good evidence that puzzles belonging to the same category can differ markedly in their difficulty, as indicated by wide variability in solution rates. For example, two rebus puzzles from Category 1 (i.e., Item 43—“long overdue”; Item 1—“feeling on top of the world”) have mean solution rates of 95.29–45.88%, respectively. This observation again supports the value of these presented norms for the effective selection and control of rebus stimuli in future studies.

**Table 2:** Normative data for 84 rebus puzzles (please refer to the text for further details).

**Item**	**Answer**	**Puzzle set and puzzle category**	**Number of correct solutions**	**Number of incorrect solutions**	Familiarity count for the solution phrase^*^	**Solution rate (percentage)**	**Mean solution time (s)**	**SD solution time (s)**	**Mean confidence rating**	**Mean insight rating**
43	Long overdue	B/1	81	4	84 [79]	95.29	8.02	3.26	90.45	64.00
44	Man overboard	B/1	80	5	84 [83]	94.11	8.94	4.65	96.29	58.51
62	Small talk	B/5	79	6	83 [78]	92.94	6.76	2.45	89.56	60.87
81	First aid	B/6	79	5	n/a	92.94	7.45	3.05	91.62	63.35
17	Four-wheel drive	A/4	77	5	78 [80]	90.59	9.55	3.79	80.84	57.87
58	Small print	B/4	77	8	81 [79]	90.59	7.50	2.97	88.60	65.73
69	Jack in the box	B/5	77	7	81 [81]	90.59	9.03	4.55	89.12	60.39
76	Wave goodbye	B/5	76	8	81 [76]	89.41	8.68	3.30	85.26	58.99
59	Go for it	B/4	75	9	81 [75]	88.21	8.33	3.42	85.24	55.61
25	Split personality	A/5	74	10	81 [80]	87.06	7.28	3.24	82.62	68.80
21	Big bad wolf	A/5	73	8	80 [81]	85.88	8.45	4.14	77.84	69.19
26	Top hat	A/5	73	11	79 [72]	85.88	7.35	3.37	74.42	65.82
31	Little sister	A/5	73	12	80 [76]	85.88	8.18	2.74	74.32	62.40
65	A big if	B/5	73	9	79 [62]	85.88	8.99	4.95	64.62	55.52
80	Upside down	B/6	73	8	81[75]	85.88	9.81	4.67	81.22	57.32
30	Broken promise	A/5	72	12	81 [82]	84.71	9.38	4.86	78.16	63.31
4	Stay overnight	A/1	71	12	75 [78]	83.52	10.48	5.72	61.34	44.68
47	London underground	B/2	70	11	81 [81]	82.35	12.26	4.97	89.35	49.86
20	Half-hearted	A/5	69	16	82 [79]	81.18	8.70	3.99	82.71	58.45
15	Too little too late	A/4	67	7	84 [84]	78.82	12.66	5.67	78.20	61.96
16	Forty winks	A/4	67	14	76 [75]	78.82	10.29	5.24	78.70	76.00
57	All for one and one for all	B/4	67	11	81 [81]	78.82	16.15	5.70	82.13	50.33
52	You're in trouble	B/3	66	14	80 [79]	77.65	12.78	6.33	81.89	49.25
2	Bend over backwards	A/1	64	13	80 [83]	75.29	11.26	5.98	76.29	52.77
35	Cross breed	A/6	64	16	77 [67]	75.29	10.69	5.02	77.58	52.60
70	Big brother	B/5	64	14	81 [81]	75.29	8.68	4.02	75.02	55.81
46	Swim underwater	B/2	63	13	78 [40]	74.12	12.42	4.47	82.59	49.25
51	Tongue in cheek	B/3	63	11	83 [80]	74.12	11.62	4.85	89.49	50.14
53	Within reason	B/3	57	10	81 [79]	67.06	12.07	6.01	82.21	56.26
39	Side show	A/6	56	26	73 [70]	65.88	9.30	5.51	67.00	55.94
60	Tricycle	B/4	52	26	77 [68]	61.18	10.62	5.09	75.38	50.10
29	Shrinking violet	A/5	50	16	65 [60]	58.82	11.55	5.45	79.98	58.80
7	Burying your head in the sand	A/3	47	18	78 [81]	55.29	15.39	8.07	73.59	42.57
49	Deep in thought	B/3	46	20	82 [77]	54.12	10.66	4.75	89.39	53.79
54	One in a million	B/3	45	16	84 [83]	52.94	12.54	5.18	91.60	58.16
38	Pretty please	A/6	44	15	82 [80]	51.76	9.08	3.23	76.72	64.89
41	Falling in love	A/6	42	23	77 [81]	49.41	12.76	5.91	69.91	47.79
55	Tripod	B/3	42	32	73 [61]	49.41	10.71	5.58	74.25	56.59
11	Walk in the park	A/3	41	20	77 [83]	48.34	12.51	5.31	81.22	44.00
37	Uphill struggle	A/6	41	33	79 [79]	48.23	9.74	3.36	70.29	57.88
23	Shop “till you drop”	A/5	40	37	81 [83]	47.06	14.91	6.17	77.91	53.40
56	More often than not	B/4	40	33	81 [76]	47.06	12.11	5.61	86.93	58.48
1	Feeling on top of the world	A/1	39	22	84 [83]	45.88	14.06	7.43	77.61	56.03
3	Dinner on the table	A/1	39	29	75 [78]	45.88	15.06	6.73	61.46	41.26
19	Forgive and forget	A/4	39	35	81 [83]	45.88	14.47	5.74	78.76	41.35
72	Tongue in cheek	B/5	38	36	82 [80]	44.71	13.54	4.98	87.21	58.16
77	Warm up	B/6	36	30	83 [78]	42.35	11.82	4.83	83.08	51.49
40	Quite right	A/6	35	35	81 [77]	41.18	11.12	6.11	80.54	57.54
8	Pie in the sky	A/3	33	39	76 [75]	39.29	10.87	4.41	78.91	45.24
33	Downwind	A/5	32	44	71 [73]	37.64	10.80	6.37	68.79	47.81
50	Singing in the rain	B/3	31	13	82 [78]	36.47	16.82	6.53	92.13	50.35
12	Bad intention	A/3	30	30	78 [71]	35.29	17.41	7.54	73.30	39.90
45	Running on empty	B/1	30	23	80 [77]	35.29	13.74	4.98	74.80	60.21
71	Growing old	B/5	29	33	78 [77]	34.12	12.62	6.52	82.93	49.07
27	Extended family	A/5	28	31	78 [82]	32.94	12.21	5.45	74.14	47.21
75	Tall order	B/5	27	41	80 [77]	31.76	10.01	5.08	75.04	57.11
18	No one to blame	A/4	25	47	78 [77]	29.41	11.92	5.79	55.80	48.04
22	Balanced diet	A/5	25	35	83 [81]	29.41	11.26	5.80	84.00	36.85
79	Waterfall	B/6	24	54	77 [76]	28.24	10.62	4.20	62.58	57.54
32	Not bad	A/5	23	36	77 [81]	27.06	10.54	6.37	53.48	52.50
5	Try to understand	A/2	22	35	80 [77]	25.88	19.93	6.58	73.22	36.54
10	Reading between the lines	A/3	21	16	85 [84]	24.71	22.07	5.22	61.13	53.22
82	What goes up must come down	B/6	20	21	82 [83]	23.53	17.52	6.46	88.30	49.60
84	Round of drinks	B/6	20	46	83 [78]	23.53	14.75	5.03	83.25	42.80
6	Foot in the door	A/3	19	28	80 [79]	22.35	18.58	6.44	76.16	26.47
13	Six of one and half a dozen	A/4	19	32	71 [66]	22.35	19.25	4.82	71.90	44.90
73	Long time no see	B/5	19	29	83 [83]	22.35	17.19	6.21	91.63	34.42
83	Search high and low	B/6	17	30	83 [80]	20.00	14.34	6.78	86.00	43.76
14	Up for grabs	A/4	16	18	80 [80]	18.82	19.49	7.65	70.17	43.44
42	Your time is up	A/6	16	44	80 [83]	18.82	14.14	7.80	68.89	63.89
64	Through thick and thin	B/5	16	48	84 [81]	18.82	13.73	7.34	91.63	52.00
48	Put it in writing	B/3	12	37	80 [71]	14.12	17.91	6.70	80.33	32.08
61	Free for all	B/4	11	66	83 [82]	12.94	10.61	2.95	80.09	41.55
78	Up to no good	B/6	10	28	84 [82]	11.76	21.89	6.91	73.60	31.20
28	Vanishing point	A/5	8	65	68 [68]	9.41	15.04	6.02	82.38	60.38
74	Unfinished symphony	B/5	8	28	57 [57]	9.41	21.30	6.69	74.88	33.63
36	Parallel bars	A/6	5	49	54 [61]	5.88	14.44	9.35	52.80	54.00
67	Break time	B/5	3	70	n/a	3.53	8.99	4.51	60.00	20.33
68	Partly cloudy	B/5	3	56	72 [66]	3.53	22.64	6.82	17.00	36.33
34	Unfinished business	A/5	1	50	80 [82]	1.18	15.02		99.00	71.00
63	Split second timing	B/5	1	50	80 [65]	1.18	13.17		100	1.00
66	Emergency stop	B/5	1	50	81 [78]	1.18	12.83		100	1.00
9	Partridge in a pear tree	A/3	0	25	77 [77]				
24	A large overdraft	A/5	0	51	53 [71]				

*Blank cells are provided where no correct answers were given for a particular rebus puzzle. For Items 67 and 81 an “n/a” response is noted for the familiarity count for these solution phrases because of a computer error in registering responses*.

* *Phrase familiarity count for the phrase in which the corresponding rebus puzzle had not been encountered*.

Table 2 also shows that the mean confidence ratings for correctly solved rebus puzzles are all above the scale mid-point of 50, with the exception of just one item (i.e., Item 68—“partly cloudy”—with a confidence score of 17). These data indicate that when participants solve a puzzle they generally have above average confidence in the correctness of the solution, although such confidence stretches across the full range above the scale midpoint from 52.80 right up to 100. When it comes to item selection for future studies using rebus puzzles then the mean confidence data could be very useful for controlling for problem characteristics (e.g., enabling mean confidence scores for puzzles to be equated across different difficulty levels).

In relation to the performance measures for rebus puzzles that are concerned with self-perceived solution strategies (i.e., analysis vs. insight), Table 2 indicates a good degree of variability in scores across the rebus puzzles, with scores ranging from 1 at the analysis end of the scale to 76 at the insight end. Interestingly, however, scores on this measure generally cluster between 35 and 65 (i.e., 15 points either side of the scale midpoint), with only a few puzzles having scores that extend beyond these lower and upper bounds. This finding suggests that *either* insight *or* analysis solution strategies may be deployed when solving a majority of these rebus items, with averaging of scores inevitably leading to the bunching of scores around the scale midpoint. We view this observation positively, as it suggests that rebus puzzles provide an excellent way to explore underpinning problem-solving processes associated with insight-based solutions vs. analysis-based solutions.

### Solution Strategies and Solution Correctness

Following on from the aforementioned point, we note that recent research has revealed that solutions to problems that are generated by a process of self-reported insight are more likely to be correct than solutions generated by a process of analysis. For example, Salvi et al. (2016) demonstrated this finding across CRA problems, anagrams, rebus puzzles and fragmented line drawings, with other researchers reporting the same effect with magic tricks (see Danek et al., 2014b; Hedne et al., 2016). In explaining this so-called “accuracy effect” in relation to insight solutions, Salvi et al. (2016; see also Danek and Salvi, 2018) propose that the effect is most likely to be attributable to the “all-or-nothing” manner in which insight solutions emerge into consciousness once non-conscious processing has been completed. In contrast, solutions that are based on analysis can be “guesses” that derive from conscious processing that is prematurely terminated, especially under time constraints. Such guesses would give rise to more errors of commission (i.e., incorrect responses) than errors of omission (i.e., timeouts) when compared to insight responses (for related evidence see Kounios et al., 2008).

In order to provide further corroboratory evidence for the existence of this consistent accuracy effect in relation to insight solutions, we applied a standard accuracy analysis to the present dataset to determine whether rebus puzzles that are solved via insight are more likely to be correct than rebus puzzles solved via analysis. Of all the solution responses designated as being based on insight (i.e., falling between 51 and 100 on the analysis/insight scale), an average of 65% (*SD* = 27) were correct. In contrast, of all the solution responses designated as being based on analysis (i.e., falling between 1 and 49 on the analysis/insight scale), an average of 54% (*SD* = 27) were correct. A paired-samples *t*-test revealed that insight solutions were indeed significantly more likely to be correct than analytic solutions, *t* = 4.76, *p* < 0.001.

Following Salvi et al. (2016), we also conducted a secondary analysis of the dataset with a narrower response window than the full 30 s that was available for solving each rebus puzzle. The analysis was similar to that just described, except that only those responses with latencies within a 2–10 s time-window were included. This approach helps to ensure a similar balance of insight and analytic responses in the dataset whilst also eliminating very fast responses made during the first 2 s, given that participants might inadvertently label these as insight-based (see Salvi et al., 2016). This revised analysis again revealed the predicted accuracy effect, with insight responses being significantly more likely to be correct (*M* = 79%, *SD* = 27) than analytic responses (*M* = 65%, *SD* = 36), *t* = 4.69, *p* < 0.001.

The previous approach to analyzing the link between solution strategies and solution correctness revolved around a dichotomous measure of solution strategies as being insight-based (above 51 on the analysis/insight scale) vs. analysis-based (below 49 on the analysis/insight scale). Conditionalizing solution correctness on solution strategy has become the standard approach in the literature for examining the existence of the accuracy effect. However, on the assumption that there is a very tight coupling between insight solutions and solution correctness it is also useful to test for the existence of a “correctness effect,” whereby correct solutions are more likely to be solved by insight than are incorrect solutions. This correctness effect should arise because of the “all-or-nothing” manner in which correct solutions typically arise via insight in comparison to the way in which analysis can promote incorrect guesses.

Determining the existence of a correctness effect involves conditionalizing self-reported solution strategies on the correctness of the proffered solution. To conduct the requisite analysis, we made use of participants' exact ratings on the 1–100 analysis/insight scale, adding a greater degree of precision to the measure of analysis vs. insight than that which would arise from simply dichotomizing the scale at its midpoint. Our resulting analysis simply applied a paired samples *t*-test to compare participants' mean solution strategy scores for all correct solutions vs. their mean solution strategy scores for all incorrect solutions. This test revealed that correct responses resulted in a significantly higher analysis/insight score (*M* = 55.74, *SD* = 22.40) than incorrect responses (*M* = 47.81, *SD* = 18.62), *t* = 4.64, *p* < 0.001. The observation that the mean analysis/insight score for correct response fell above the scale midpoint indicates a more insight-based solution strategy for correct solutions. In contrast, the observation that mean analysis/insight score for incorrect response fell below the scale midpoint indicates a more analysis-based solution strategy for incorrect solutions.

In sum, when considered together, the full set of analyses of the relation between solution strategies and solution correctness indicates a tight, bidirectional relationship in the form of both an accuracy effect (insight solutions are more likely to be correct that analytic solutions) and a correctness effect (correct solutions are more likely to be insight-based than incorrect solutions).

### Solution Strategies, Solution Correctness, and Solution Confidence

In considering potential explanations of the accuracy effect for insight solutions, Danek and Salvi (2018) contemplate the viability of an account based on the notion that solvers might use their confidence in accurate responses as a metacognitive cue for reporting the solution as being based on insight. The essential idea here is that when accurate, solvers might feel highly confident about their solution and therefore retrospectively report having had an insight experience. As Danek and Salvi (2018) acknowledge, at first glance this account of the accuracy effect seems to gain support from the observation that confidence correlates highly with insight ratings (Webb et al., 2016; Danek and Wiley, 2017). However, Danek and Salvi (2018) counter that the studies that reveal a correlation between confidence and insight specifically mention “confidence” in their instructions to participants, possibly inflating the observed correlation. Moreover, they note that solvers sometimes also feel confident about *incorrect* solutions (Danek and Wiley, 2017), suggesting that it is unlikely that the accuracy effect is solely based on high confidence serving as a metacognitive cue for insight ratings.

We agree with Danek and Salvi's cautionary arguments and consider that a causal link between confidence judgments and insight ratings seems unlikely. Given that the present study elicited confidence ratings from participants for all generated solution responses, we analyzed the present dataset with a view to shedding further light on how solution confidence is related to solution strategy and solution correctness. A 2 × 2 Analysis of Variance (ANOVA) was conducted to determine the difference in confidence ratings according to solution correctness (correct vs. incorrect) and solution strategy (insight vs. analysis—again based on dichotomized scores).

The ANOVA revealed that there was no main effect of solution strategy, with confidence ratings for solutions generated via insight (*M* = 59.56, *SE* = 1.62) not differing significantly from confidence ratings for solutions generated via analysis (*M* = 57.46, *SE* = 1.41), *F*_(1, 136)_ = 1.47, *MSE* = 409.40, *p* = 0.23. There was, however, a significant main effect of solution correctness, with confidence ratings being significantly higher for correct solutions (*M* = 74.35, *SE* = 1.56) in comparison to incorrect solutions (*M* = 42.68, *SE* = 1.58), *F*_(1, 136)_ = 273.28, *MSE* = 502.92, $\eta_{\text{p}}^{2}$ = 0.67, *p* < 0.001. There was no solution strategy by solution correctness interaction, *F* < 1, *p* = 0.42. These results support the existence of heightened confidence for correct solutions over incorrect solutions whether or not the problem was solved via insight, suggesting that there is no unique and clear-cut link between perceived confidence and insight phenomenology, thereby supporting the arguments of Danek and Salvi (2018).

### Solution Strategies, Solution Correctness, and Response Time

A 2 × 2 ANOVA was also conducted to determine the difference in mean solution times as a function of solution correctness (correct vs. incorrect responses) and solution strategy (insight vs. analysis). Given that solution-time data are often found to be positively skewed, thereby undermining the assumptions required for the pursuit of parametric data analysis, we first determined the skew in the dataset for each condition according to each set of rebus puzzles. We observed that two conditions demonstrated positive skew in their associated solution-time data, with skew values (i.e., 1.92 and 1.74) above typically accepted levels (e.g., Tabachnick and Fidell, 2013). As a result, a Log_10_ transformation was performed on the solution-time data for all conditions prior to running the ANOVA (see Table 3 for the natural and Log_10_ mean solution times for each condition).

**Table 3:** Mean natural solution times (s) and mean Log_10_ solution times as a function of solution strategy (insight vs. analysis) and solution correctness (correct vs. incorrect).

	**Puzzles solved by insight**		**Puzzles solved by analysis**	
	**Correct**	**Incorrect**	**Correct**	**Incorrect**
Mean Natural Solution Times	10.93 (4.12)	15.76 (6.19)	13.32 (4.50)	18.20 (5.79)
Mean Log_10_ Solution Times	1.01 (0.15)	1.16 (0.18)	1.10 (0.15)	1.23 (0.17)

*Standard deviations are shown in parenthesis*.

For the transformed solution-time data the ANOVA revealed a significant main effect of solution strategy, with problems solved via insight being solved significantly faster (*M* = 1.08, *SE* = 0.01) than problems solved via analysis (*M* = 1.17, *SD* = 0.01), *F*_(1, 136)_ = 46.04, *MSE* = 0.02, $\eta_{\text{p}}^{2}$ = 0.25, *p* < 0.001. This finding underscores how analysis is often a more laborious process than insight. There was a significant main effect of solution correctness, with mean solution times being significantly faster for correct responses (*M* = 1.06, *SE* = 0.01) in comparison to incorrect responses (*M* = 1.20, *SE* = 0.01), *F*_(1, 136)_ = 217.47, *MSE* = 0.01, $\eta_{\text{p}}^{2}$ = 0.61, *p* < 0.001. This observation is unsurprising given that correct solutions are more likely to arise from a (fast) insight process than incorrect solutions. There was no solution strategy by solution correctness interaction, *F*_(1, 136)_ = 0.79, *MSE* = 0.01, *p* = 0.38.

### Phrase Familiarity

In Table 2, we also provide two familiarity counts for the solution phrase that was associated with each rebus puzzle, with each familiarity count having a maximum value 85, in line with the number of participants tackling each set of rebus puzzles. The importance of providing two familiarity counts for each particular solution phrase is to draw a distinction between a familiarity rating given to a solution phrase after the participant had encountered the corresponding rebus puzzle, compared to having not encountered the corresponding rebus puzzle. This distinction is made possible by the fact that each participant rated the familiarity for each of the 84 solution phrases, whilst only having attempted to solve 42 of the rebus puzzles relating to these phrases. The first familiarity count presented in Table 2 is for the solution phrase from the set in which the corresponding rebus puzzle had been encountered. The second familiarity count (provided in square brackets in Table 2) is for the solution phrase from the set in which the corresponding rebus puzzle had not been encountered.

An independent samples *t*-test was conducted to determine if there was a significant difference between these two familiarity counts. This analysis revealed that phrase familiarity (*M* = 78.61, *SD* = 5.98) was significantly higher when the rebus puzzle corresponding to the solution phrase had been encountered in comparison to when the rebus puzzle corresponding to the solution phrase had not been encountered (*M* = 76.41, *SD* = 7.43), *t* = 2.08, *p* = 0.039. This suggests that there might be a small but reliable bias toward a judgment of familiarity being given for a solution phrase for which the previous rebus puzzle had been encountered, even though the “correct” solution phrase for each rebus has not been provided.

The familiarity data for solution phrases enabled us to explore a number of potentially interesting associations between phrase familiarity and the performance measures identified in Table 2. These associations were explored using the item-based performance data (i.e., frequency counts and mean scores) for the 84 rebus puzzles that are depicted in Table 2. In order to explore patterns of association involving the familiarly data, we took the two familiarity count measures previously identified and transformed them into percentage familiarity scores. To reiterate, the first familiarity score was for the rating of a solution phrase from the set in which the corresponding rebus puzzle had been encountered. The second familiarity score was for the rating of the solution phrase from the set in which the corresponding rebus puzzle had not been encountered. Having computed the two percentage familiarity scores for each rebus puzzle we then correlated these independently with five performance measures for each rebus item, that is: its solution rate, its error rate (i.e., the percentage of incorrect solutions), the mean confidence in correct solutions, the mean analysis/insight score for correct solutions and the mean response time (seconds) for correct solutions.

Pearson correlation coefficients indicated that each familiarity score was not significantly associated with the solution rate (*r* = 0.10 and *r* = 0.05, respectively, both *p*s > 0.05). The absence of an association between phrase familiarity and solution success attests to the challenging nature of many of the rebus puzzles despite the fact that the underpinning solution phrase was well-known. For example, the two rebus puzzles with a 0% solution rate (Item 24—“large overdraft”; Item 9—“partridge in a pear tree”) still received scores of over 50% for the familiarity of their solution phrases. In other words, even when there is a good degree of familiarity with the underpinning solution phrase for a rebus puzzle, this does not necessarily translate into the ability to solve the rebus puzzle.

In terms of other observed associations, there was a weak but nevertheless significant negative correlation between the first familiarity rating (when the rebus puzzle corresponding to that particular solution phrase had been encountered) and error rate (*r* = −0.24, *p* = 0.03), indicating that as familiarity with the underpinning solution phrase increased, the percentage of incorrect solutions decreased. A similar pattern was found for the second familiarity measure (*r* = −0.16), but this failed to reach significance. Neither of the phrase familiarity scores was significantly associated with participants' mean confidence in correct rebus solutions (*r* = 0.20 and *r* = 0.14, respectively), with their mean analysis/insight scores for correct solutions (*r* = 0.12 and *r* = 0.16) or with their mean response time for correct solutions (*r* = 0.10 and *r* = 0.12), all *p*s > 0.05.

### Rebus Puzzle Categories

As discussed in the materials section, rebus puzzles were divided into six categories according to common solution principles (refer to Table 1 for the distribution of categories across rebus puzzle Set A and Set B). The measurements presented in Table 2 are reorganized in Table 4 so as to show data collapsed across the six rebus categories. In other words, these reconfigured data provide an indication of how each of the dependent variables differs according to each particular rebus puzzle category.

**Table 4:** Normative data for each of the six rebus puzzle categories.

**Puzzle category**	**Mean number of correct solutions**	**Mean number of incorrect solutions**	**Mean familiarity count for solution phrases**	**Mean solution rate (Percentage)**	**Mean solution time (s)**	**SD solution time (s)**	**Mean confidence rating**	**Mean insight rating**
1. A word, picture or number, over another word, picture or number	57.71	15.42	80.29	67.89	11.65	5.54	76.89	53.92
2. A word, picture or number, under another word, picture or number	51.67	19.67	79.67	60.78	14.87	5.34	81.72	45.22
3. A word presented within another word	36.87	21.93	79.73	44.48	14.42	5.92	80.40	47.00
4. A play on words with numbers	48.62	23.92	79.38	57.19	12.53	4.96	77.90	53.49
5. Imagery	38.50	30.63	76.76	46.86	11.68	5.05	77.93	48.19
6. Spatial	36.38	28.94	78.40	42.79	12.47	5.51	75.84	53.22

The data in Table 4 indicate that rebus puzzles in Categories 1, 2, and 4 gave rise to higher mean solution rates than those in Categories 3, 5, and 6, suggesting that the spatial and imagery related rebus puzzles are generally more challenging than those related to words, with the exception of the “word presented within another word” puzzles (Category 3), which are also more difficult than the other word-related rebus items. Nevertheless, the item-based data presented in Table 2 reveal considerable variability in difficulty levels for items within each of the categories, ensuring that item selection in future studies can capitalize on such variability in situations where a puzzle-difficulty manipulation is a desirable feature of an experimental design.

With respect to mean analysis/insight ratings, the descriptive data in Table 4 indicate very limited variability in ratings across the different rebus categories, with mean analysis/insight scores showing a narrow range from 45.22 to 53.22. A similar picture of homogeneity emerges for: (1) mean confidence ratings, which again show considerable similarity across categories, ranging from 75.84 to 81.72; and (2) mean solution times, which range from 11.65 to 14.87 s. Such high levels of similarity in people's performance measures across rebus categories support the usefulness of the present norming data to inform item selection for future studies.

We finally note that the unequal number of rebus puzzles in each of the rebus categories (including the particularly low number of puzzles in Categories 1 and 2) precludes the pursuit of formal, inferential analysis of the possible performance differences that might arise across rebus categories.

## General Discussion

Classic insight problem-solving tasks such as the candle problem (Duncker, 1945), two-string problem (Maier, 1930) and nine-dot problem (Maier, 1930) are complex and time-consuming to solve whilst also yielding potentially ambiguous solutions and being susceptible to the effects of confounding variables (cf. Ball et al., 2015). Furthermore, given the popularity of these tasks in the problem-solving literature and their exposure in research, the solutions to classic insight problems are often generally well-known. This has led to the advent of additional pools of insight-based problems, such as CRAs (e.g., Bowden and Jung-Beeman, 2003b; Wu and Chen, 2017), magic tricks (e.g., Danek et al., 2014a,b) and rebus puzzles (e.g., MacGregor and Cunningham, 2008, 2009; Salvi et al., 2015).

The use of both CRAs and rebus puzzles is especially appealing, since in contrast to classic insight problems they are relatively simpler and yield unambiguous single-word answers (CRAs) or single phrases (rebus puzzles). They are easy to administer to participants and straightforward to record answers for and they are additionally relatively fast for solvers to generate solutions to. Moreover, multiple problems can be presented within a single session to maximize the number of observations per experimental condition, and therefore the reliability of the data obtained. The problems are also well-suited to study using fMRI (e.g., Kizilirmak et al., 2016) and EEG (e.g., Li et al., 2016) due to their simplicity and possibility for presentation within a compressed visual space. However, the utility of these insight problems in research is heavily dependent upon the knowledge of baseline problem difficulties and solution times (i.e., normative data).

In addition to the many positive features of CRAs and rebus puzzles that we have identified, we also note that they appear to share with classic insight problems the same kinds of underpinning component processes and phenomenological experiences (Bowden and Jung-Beeman, 2003a, 2007). For example, both CRAs and rebus puzzles have the potential to engender initial misdirection along ineffective solution avenues or the failure of effective retrieval processes that can culminate in impasse and a subsequent “Aha!” experience when a route toward a solution suddenly comes to mind (Salvi et al., 2015). Therefore, both CRAs and rebus puzzles can be used to address the degree to which participants differ in their tendency toward solving particular items via insight or analytic strategies.

The extant literature provides extensive normative data for CRAs, which have been normed for participant samples in the USA (Bowden and Jung-Beeman, 2003b), the UK (Sandkühler and Bhattacharya, 2008), China (Wu and Chen, 2017), and Italy (Salvi et al., 2016). To the best of our knowledge, however, there are very limited normative data for rebus puzzles, with the only data that are currently available being restricted to a set of 88 Italian rebus puzzles (Salvi et al., 2015). Due to the linguistically contextualized nature of rebus puzzles, however, it is important to extend the base of normative data for such problems to other languages, including UK English. In setting out to address this gap in the literature we endeavored to undertake a norming study with a set of carefully-selected rebus puzzles for which we could obtain data relating to solution rates, error rates, solution times, solution confidence, self-reported solution strategies (insight vs. analysis), and familiarity with the solution phrases.

In Table 2, we provide normative data for each of the 84 rebus puzzles that we examined, which were assessed as two separate sets of 42 puzzles. Within Table 2, the data are depicted in descending order of their mean solution rate within the 30 s time limit available. Also reported in Table 2 are the number of incorrect solutions, classified as attempts at a response that gave rise to incorrect words or phrases. Mean solution times (and standard deviations) are also displayed. Since rebus puzzles may differentially engender insight vs. analytic solution strategies, we additionally report data for participants' self-reported solution strategies. In Table 4, we provide normative data for rebus puzzles as a function of the rebus category within which they fell in terms of the underpinning solution principle. In Appendices A, B in Supplementary Material, all rebus puzzles are presented pictorially according to their presented set.

Since solutions to rebus puzzles are contingent on knowledge of the particular solution phrase underpinning the problem, we thought it critical to report data on the familiarity of each phrase that comprised a rebus solution. We observed that participants were largely familiar with the rebus solution phrases presented to them. Therefore, we can be confident that the rebus puzzles that were normed in the present study relate to well-known UK English phrases or sayings. We distinguished between two types of familiarity with the solution phrases, and found that the familiarity for a solution phrase in which the corresponding rebus puzzle had been attempted was significantly higher than the familiarity for a solution phrase in the absence of previously encountering the associated rebus puzzle. It is interesting to note that this bias existed even though the “correct” solution phrase for each rebus was not directly provided to the participants. The mere exposure to the associated rebus puzzle appeared to increase a subsequent familiarity rating for the solution phrase. Neither familiarity rating was associated with solution rate, mean confidence, mean insight or mean response time. Familiarity ratings were, however, associated with the percentage of incorrect solutions, in that greater familiarity led to fewer incorrect solutions, although this association was restricted to the familiarity rating for solution phrases for which the corresponding rebus puzzles had been attempted. The absence of significant associations between phrase familiarity and solution rate, mean confidence, mean insight and mean response time are unsurprising, given that we observed generally high familiarity levels for most of the rebus puzzle solution phrases.

More detailed analyses of the present dataset were also undertaken, which provide further support for a growing body of evidence demonstrating that solutions that arise from a self-reported insight process are more likely to be correct than solutions that arise via a process of analysis (e.g., Metcalfe, 1986; Salvi et al., 2015; Danek and Salvi, 2018). This particular advantage for insight responses appears to hold not just for rebus puzzles, but also for CRA problems, magic tricks and anagrams (Danek and Salvi, 2018). Not only are insight solutions more likely to be correct than analytic solutions, they also arise more rapidly. However, these particular “insight” advantages were not seen to extend to people's self-rated confidence in solutions that were generated via insight (see also Hedne et al., 2016; Salvi et al., 2016.

We suggest that the rich seam of norming data reported here for rebus puzzles can be tapped to create different sets of stimuli that are closely matched on critical variables such as problem difficulty. This matching can be done either by hand, or preferably, via the use of stimulus matching software programs such as “Match” (Van Casteren and Davis, 2007) that automate the selection of groups of stimuli sets from larger pools through matching on multiple dimensions. In relation to the issue of controlling stimulus selection, it is also necessary to consider the structure of rebus puzzles and the resulting strategy that might be adopted to solve a particular problem. As noted in our method section (see also Salvi et al., 2015), given the structural similarity of some rebus puzzles, care must be taken to separate these problems to control for, or minimize, order and carry-over effects from one problem to subsequent ones. This is important when presenting a set of problems either within or between experimental blocks. That is, the solution for one problem with a particular structure (e.g., spatial), may influence the finding of a solution for a later encountered problem with a similar structure (e.g., via transfer or priming effects).

This latter issue is apparent if we consider Item 49 (“THODEEPUGHT”; solution: “deep in thought”) and Item 51 (“CHTONGUEEK”; solution: “tongue in cheek”). Here we see an example of two different problems from Category 3 (i.e., a word within a word), where the rebus is structured in such a way that the first word is quite literally presented “within” another word. Our categorization of the problems into different structural types that were validated through interrater reliability checks, can be used to help researchers to identify such overlap in rebus puzzles and thus avoid an issue of presenting problems underpinned by a similar structure or solution strategy. It remains unknown to what extent the transfer of problem structures assists solution rates or solution times for rebus puzzles from common categories. The present dataset does not permit an analysis of order effects according to each rebus puzzle within each category. However, descriptive statistics provided for each rebus puzzle do demonstrate a broad range of solution rates and solution times—even for problems within the same puzzle category—which is suggestive of minimal practice effects. Drawing on an example of two rebus puzzles from Category 3, solution rates for these two puzzles varied from 74.12 to 14.12%.

In conclusion, we hope that the materials and normative data presented here will arm researchers with important apparatus through which problem solving and creativity can be studied with UK English speaking participants. Like CRAs and their conceptual antecedents, RATs, rebus puzzles can be used across a broad range of domains to study problem solving and creative thinking, affect, psychopathologies and metacognitive processes.

## Author Contributions

All authors listed have made a substantial, direct and intellectual contribution to the work, and approved it for publication.

### Conflict of Interest Statement

The authors declare that the research was conducted in the absence of any commercial or financial relationships that could be construed as a potential conflict of interest.
